# Supporting People With Type 2 Diabetes in the Effective Use of Their Medicine Through Mobile Health Technology Integrated With Clinical Care to Reduce Cardiovascular Risk: Protocol for an Effectiveness and Cost-effectiveness Randomized Controlled Trial

**DOI:** 10.2196/32918

**Published:** 2022-02-21

**Authors:** Andrew Farmer, Louise Jones, Nikki Newhouse, Cassandra Kenning, Nicola Williams, Yuan Chi, Y Kiera Bartlett, Catrin Plumpton, Jenny McSharry, Rachel Cholerton, Emily Holmes, Stephanie Robinson, Julie Allen, Bernard Gudgin, Carmelo Velardo, Heather Rutter, Rob Horne, Lionel Tarassenko, Veronika Williams, Louise Locock, Rustam Rea, Ly-Mee Yu, Dyfrig Hughes, Peter Bower, David French

**Affiliations:** 1 Nuffield Department of Primary Care Health Sciences University of Oxford Oxford United Kingdom; 2 Centre for Primary Care and Health Services Research University of Manchester Manchester United Kingdom; 3 Institute of Biomedical Engineering Department of Engineering Science University of Oxford Oxford United Kingdom; 4 Manchester Centre for Health Psychology University of Manchester Manchester United Kingdom; 5 Centre for Health Economics and Medicines Evaluation Bangor University Bangor United Kingdom; 6 Health Behaviour Change Research Group, School of Psychology National University of Ireland Galway Galway Ireland; 7 Patient Advocate Nuffield Department of Primary Care Health Sciences University of Oxford Oxford United Kingdom; 8 Sensyne Health plc Oxford United Kingdom; 9 Oxford University Hospitals National Health Service Foundation Trust Oxford United Kingdom; 10 Centre for Behavioural Medicine University College London London United Kingdom; 11 School of Nursing Nipissing University North Bay, ON Canada; 12 Health Services Research Unit University of Aberdeen Aberdeen United Kingdom; 13 Oxford Centre for Diabetes, Endocrinology and Metabolism Oxford University Hospitals National Health Service Foundation Trust Oxford United Kingdom; 14 NIHR Oxford Biomedical Research Centre Oxford University Hospitals National Health Service Foundation Trust Oxford United Kingdom

**Keywords:** diabetes, SMS text messages, cardiovascular risk prevention, medication adherence, digital health, randomized controlled trial

## Abstract

**Background:**

Type 2 diabetes is a common lifelong condition that affects over 400 million people worldwide. The use of effective medications and active self-management can reduce the risk of serious complications. However, people often have concerns when starting new medications and face difficulties in taking their medications regularly. Support provided by brief messages delivered through mobile phone–based SMS text messages can be effective in some long-term conditions. We have identified promising behavior change techniques (BCTs) to promote medication adherence in this population via a systematic review and developed SMS text messages that target these BCTs. Feasibility work has shown that these messages have fidelity to intended BCTs, are acceptable to patients, and are successful in changing the intended determinants of medication adherence. We now plan to test this intervention on a larger scale in a clinical trial.

**Objective:**

The aim of this trial is to determine the effectiveness and cost-effectiveness of this intervention for reducing cardiovascular risk in people with type 2 diabetes by comparing it with usual care.

**Methods:**

The trial will be a 12-month, multicenter, individually randomized controlled trial in primary care and will recruit adults (aged ≥35 years) with type 2 diabetes in England. Consenting participants will be randomized to receive short SMS text messages intended to affect a change in medication adherence 3 to 4 times per week in addition to usual care. The aim is to test the effectiveness and cost-effectiveness of the intervention when it is added to usual care. The primary clinical outcome will be a composite cardiovascular risk measure. Data including patient-reported measures will be collected at baseline, at 13 and 26 weeks, and at the end of the 12-month follow-up period. With 958 participants (479 in each group), the trial is powered at 92.5% to detect a 4–percentage point difference in cardiovascular risk. The analysis will follow a prespecified plan. A nested quantitative and qualitative process analysis will be used to examine the putative mechanisms of behavior change and wider contextual influences. A health economic analysis will be used to assess the cost-effectiveness of the intervention.

**Results:**

The trial has completed the recruitment phase and is in the follow-up phase. The publication of results is anticipated in 2024.

**Conclusions:**

This trial will provide evidence regarding the effectiveness and cost-effectiveness of this intervention for people with type 2 diabetes.

**Trial Registration:**

ISRCTN Registry ISRCTN15952379; https://www.isrctn.com/ISRCTN15952379

**International Registered Report Identifier (IRRID):**

DERR1-10.2196/32918

## Introduction

### Type 2 Diabetes and Medication Adherence

Type 2 diabetes is a lifelong condition that can cause serious long-term health problems [1]. It is one of the most common long-term conditions, affecting 422 million people worldwide [2] and 4.7 million people in the United Kingdom [3]. It can lead to major complications, including cardiovascular disease (CVD), renal failure, and neuropathy [1]. The global economic burden of diabetes is projected to reach up to 2.2% of the global gross domestic product [4], and many of these costs are because of preventable complications. Treatments to reduce risks of complications from type 2 diabetes are effective if taken as intended [5,6]. However, concerns about medicines and difficulties in taking them regularly, whether intentional or unintentional, are common [7]. The cost of nonadherence to diabetes medication in the United Kingdom has been estimated at £100 million (US $135 million) per year in avoidable treatment costs alone [8].

Some services, such as pharmacy medication checks, use of blister packaging, written reminders, and routine education, are available to support people in taking their medication regularly, but evidence of their effectiveness and cost-effectiveness is not strong [9]. These services may not be right for everyone and are often targeted at particular groups or designed as *one-off* services. Understanding and improving this situation could make a major contribution to health.

### Digital Health Interventions and Brief Messaging

Systematic reviews of SMS text messaging to support adherence to treatment and of mobile health interventions in diabetes have identified some effective interventions [10,11]. There are a few trials testing the impact of brief messaging in type 2 diabetes, but they do not test systematically developed interventions, and many are at risk of bias or have short-term follow-up. However, despite variation in response in different settings and differences in trial design, studies to date have not resolved continuing uncertainty about implementation in routine health care [10,11]. Trials of SMS text messaging for preventing cardiovascular risk and lowering blood pressure have shown clinically relevant changes in outcomes compared with usual care [12,13].

There is substantial evidence that personalized interventions are more effective than generic interventions [14]. Tailored interventions may be seen by recipients as more personally relevant, so they will be more likely to attend to, read, understand, and act on them. In addition, tailored interventions are designed to change the determinants of the target behavior that are relevant to individuals or to small subgroups of individuals; they therefore more precisely target the determinants of the behavior of individuals.

### Preliminary Studies

Support Through Mobile Messaging and Digital Health Technology for Diabetes (SuMMiT-D) is a program of work composed of three phases: formative work; a feasibility trial; and a large-scale, pragmatic randomized controlled trial of a mobile phone–based system. The program is intended to develop and evaluate brief, tailored behavior change messages for people with type 2 diabetes, intending to encourage regular use of diabetes medication and persistence, and modify risk factors including glucose, blood pressure, and cholesterol levels, and thus the risk of adverse outcomes, including CVD. The intervention is intended to focus on a broad range of individuals with type 2 diabetes, but those with younger onset diabetes and using insulin alone were not included, as these features often require different care pathways.

In the formative work for this trial, we identified theoretical constructs and features of intervention content found to be associated with medication adherence in people with type 2 diabetes [15] and mapped them onto a standard taxonomy of behavior change techniques (BCTs), that is, active components of interventions used to promote behavior change [16,17]. Development work aimed to ensure that the overall approach was acceptable to people with type 2 diabetes [15]. We then developed a large set of messages to target each BCT [18], through an expert consensus process and surveys with experts and patients to select messages that had fidelity to the intended BCTs and were acceptable to patients. We carried out a feasibility study and further qualitative work [18,19] and confirmed that the intervention and trial processes were acceptable and feasible [20,21] and that the responses to specific messages matched the response observed in the formative work [22].

### Aims

The primary objectives of the main SuMMiT-D trial are to determine the effectiveness and cost-effectiveness of this intervention in reducing cardiovascular risk for people with type 2 diabetes compared with usual care. In addition to the primary objectives to determine the effectiveness and cost-effectiveness, a process evaluation will provide information to further develop and refine the intervention, to explore how it can achieve a wide reach, and to explore how it can be incorporated and embedded in health care pathways. It will also further identify the precise psychological mechanisms of action through which the intervention might change behavior.

## Methods

### Overview

The SuMMiT-D trial protocol is reported according to the Standard Protocol Items: Recommendations for Interventional Trial (Multimedia Appendix 1) and the European Society for Patient Adherence, Compliance, and Persistence Medication Adherence Reporting Guidelines recommendations [23,24].

### Patient and Public Involvement

Patient members of the public are integral to this trial. A panel of 11 patient and public involvement (PPI) members with type 2 diabetes was set up for the formative stage of this program of work and continues to inform our work, reviews all patient documentation and research findings, and supports the development and refinement of the intervention.

All patient-facing documents for the SuMMiT-D trial, including the participant information sheet, informed consent form, posters, user guides, and website, were reviewed by PPI panel members. The results of the study will be made available to participants of the trial, PPI panel members, and participating general practices on the trial website.

### Research Design

The SuMMiT-D main trial is a primary care–based, 2-arm, parallel group, individually randomized controlled trial with a health economic analysis and an embedded process evaluation. The trial aims to recruit 958 participants from up to 100 general practice sites in England. Participants with type 2 diabetes who provide consent will be randomly allocated to receive individually tailored mobile phone–based messages alongside usual care (intervention) or to usual care alone (usual care).

A process evaluation will be carried out in line with the Medical Research Council (MRC) guidance on process evaluations [25] and will focus on (1) mechanisms (or theory) of change of the intervention, that is, how the intervention produces change in participants and (2) the impact of context on how the intervention works.

An economic analysis will be conducted from the perspective of the National Health Service (NHS) and Personal Social Services. The analysis will be informed by routine and self-report data and will estimate the incremental cost-effectiveness ratio expressed as the cost per quality-adjusted life year (QALY) gained.

### Setting

The trial will be conducted in general practices in England. The recruitment of practice sites will be monitored to ensure geographical spread, to include a range of sites with levels of deprivation matching the wider distribution, and to align recruitment with the burden of diabetes in the community.

### Intervention

Participants assigned to the intervention group will receive brief health-related SMS text messages based on a systematic review of the evidence identifying determinants of medication-taking behavior [17]. Messages were developed based on a systematic review of the evidence by experts [17] and refined in an iterative process of ensuring acceptability based on patient feedback and demonstration of fidelity to intended behavior change determinants, as rated by an independent group of experts [18]. A more detailed description of the intervention is given in the template for intervention description and replication checklist [26], included in Multimedia Appendix 1. Examples of messages are given in Multimedia Appendix 2 along with the corresponding BCT that the message is intended to target based on the formative work for the trial [18].

The intervention is a digital health system with the following components:

1. Participants will be sent up to 4 automated SMS text messages per week, with an average frequency of 3 per week, related to diabetes management and the use of medicine.
2. The library of SMS text messages uses different groups of BCTs (see supplementary material) to target health-related behavior changes related to the use of medicines, as well as messages targeting other aspects of diabetes care (including diet and exercise).
3. The frequency of messages received using a particular group of BCTs can be modified based on a participant’s response to individual messages by sending an SMS text message in response to a particular message asking for that type of message to be sent more or less often. Participants may incur a cost for sending messages in response depending on their network plan.
4. The style of messages is patient-centered and encourages patients to seek further relevant information (including the use of links where it is possible to select external websites; eg, Diabetes UK).

### Outcomes

The primary outcome will be a composite cardiovascular outcome adapted from the equations used for the United Kingdom Prospective Diabetes Study (UKPDS) risk engine [27]. We will evaluate the effect of changes in metabolic outcomes (glucose, blood pressure, and cholesterol levels) on the estimated risk of CVD. We will calculate CVD risk at baseline and at follow-up using the UKPDS risk engine [27]. The UKPDS risk engine is type 2 diabetes–specific and is based on 4540 patients from the UKPDS trial (1977 to 1991). It includes glycated hemoglobin (HbA_1c_) as a continuous variable and calculates the risk of developing a new coronary heart disease event.

Secondary outcomes will include glycemic control (HbA_1c_), blood pressure, total and high-density lipoprotein cholesterol (mean and clinically relevant change), self-reported smoking status, resource use, and EuroQol 5-dimension, 5-level (EQ-5D-5L). Participants will be assessed at 13, 26, and 52 weeks (not all measures at all time points), with all measurements and data being collected directly from the participants or via their medical records.

Medication adherence outcomes for antidiabetic medication will be prespecified as a proportion of participants with ≥80% medication available over 1 year, a continuous measure of the proportion of medication available over 1 year defined as the medication possession ratio (MPR), and persistence with a medication calculated from routine electronic health data [28,29]. We will also measure the MPR for statins and blood pressure–lowering medications.

### Procedures and Assessments

Potential participants who express interest in taking part in the trial will be screened by the trial team and will provide consent and submit their baseline questionnaires either on the web or on paper according to their preference. Participants will be randomized by the trial team and will receive messages for 52 weeks from randomization to the final follow-up. All participants will be asked to complete questionnaires at baseline, 13 weeks, 26 weeks, and at the end of their 52-week follow-up period. Medical note reviews will be conducted at baseline and 12 months after randomization.

### Recruitment

Potential participants will be identified through general practices in the United Kingdom. A short information leaflet will be provided to potential participants using a variety of methods, including posts displayed in waiting areas at participating general practices and given to patients by a practice team member.

Health care professionals will screen their type 2 diabetes clinic lists for identifying potentially eligible patients and invite them to participate in the study. Searches and screening may be performed periodically to enable newly potentially eligible patients to be invited. Potentially eligible patients may be contacted up to three times (by phone, letter, email, or SMS text message).

### Expressions of Interest

People interested in taking part in the trial can send their full name by SMS text messages to the trial team to register their interest. If potential participants have any difficulties in registering their interest in the trial in this way, they will be able to call a trial telephone number and will receive support in registering as required.

### Screening Assessment

Following an expression of interest, a member of the trial team will contact the potential participant by phone to provide further information about the trial and conduct screening and eligibility.

### Inclusion Criteria

The inclusion criteria for eligible participants are as follows:

- Are aged ≥35 years
- Are taking oral glucose-lowering treatment, blood pressure–lowering treatment, or lipid-lowering treatment either alone or in combination
- Have access to a mobile phone and are able, if necessary, with help (eg, relatives, friends, or neighbors), to send, understand, and retrieve brief SMS text messages in the English language

Participants who are using insulin treatment without concomitant use of oral glucose-lowering treatment; who are pregnant, within 3 months postpartum or planning pregnancy during the trial; have a serious medical condition that, in the opinion of the investigator, makes them ineligible; have been admitted to hospital within the last 3 months for hyperglycemia or hypoglycemia; or who use a pharmacist-managed monitored dosage system are ineligible.

### Informed Consent

Participants will provide consent either on the web or on paper.

### Baseline and Follow-Up Assessments

Questionnaires will be administered (on the web or by post) at the baseline assessment, at 13 weeks and 26 weeks after randomization, and at 52 weeks. The measures and schedules are detailed in Table 1.

**Table 1:** Schedule of trial outcomes and measures.

Procedures	Visits or data collection time points					
	Screening^a^	Participant expression of interest^b^	13 weeks	26 weeks	52 weeks	Any time point
Screening	✓					
Eligibility assessment		✓				
Informed consent		✓				
Demographics and additional information questionnaire (to include age, gender, and postcode)		✓				
MARS^c^ self-report scale—questionnaire		✓			✓	
EQ-5D-5L^d^ health status—questionnaire		✓	✓	✓	✓	
Health care use record questionnaire		✓			✓	
Hypothesized mediators of behavior change and technology acceptance questionnaire		✓			✓	
Brief hypothesized mediators of behavior change and technology acceptance questionnaire			✓	✓		
Brief attitudes to diabetes and treatment		✓			✓	
Data collection (including medical history and concomitant medication)		✓			✓	
Randomization		✓				
Text messaging system registration		✓				
Sending of intervention or control messages initiated		✓				
Scheduled and unscheduled contacts		✓	✓	✓	✓	
Adverse event assessments						✓
Routinely collected data^e^						✓

^a^General practitioner to screen list before mail out.

^b^Day expression of interest received or as soon as possible thereafter.

^c^MARS: Medication Adherence Report Scale.

^d^EQ-5D-5L: EuroQol 5-dimension, 5-level.

^e^Routinely collected data: hospital episode statistics from National Health Service Digital, data from medical records for metabolic outcomes, use of primary care services, and medicine costs including drug prescriptions issued.

At the baseline assessment, questionnaires will be the Medication Adherence Report Scale (MARS) [28]; EQ-5D-5L [30], a measure to assess the hypothesized mediators of effect based on developmental work and the technology acceptance model [31]; a resource use questionnaire [8]; and a brief measure of satisfaction with diabetes treatment (Multimedia Appendix 1). Experience of diabetes education, presence of a caregiver and their role in medication administration, duration of diabetes, time since last change in type 2 diabetes medication, if the pharmacy used by the participant automatically requests patients’ medication from surgery, self-reported level of education, smoking, age, gender, ethnicity, postcode, NHS number, date of birth, previous use of mobile phones and computers, and details of existing mobile phone, including contract type, will also be recorded.

Follow-up will last for 52 weeks after randomization. The following questionnaires will be completed at 13 and 26 weeks (range ±4 weeks) following randomization: EQ-5D-5L [30] and a brief questionnaire based on the health psychology theory and the technology acceptance model [31].

At 52 weeks (range ±4 weeks) following randomization, the following questionnaires will be completed: MARS, a self-report scale [32]; the EQ-5D-5L [30]; a measure based on the health psychology theory and the technology acceptance model [27]; a health care use record [8]; and a brief measure of satisfaction with diabetes treatment. A full schedule of the measures is shown in Table 1.

### Additional Trial Procedures

All participants will receive non–health-related SMS text messages at a frequency of approximately 1 every 4 weeks. These messages will be used to maintain contact and prompt completion of questionnaires. The sending and receipt of messages by mobile phones will be monitored throughout the trial and contact will be made with the participants if problems are identified.

### Randomization

Participants will be randomized after consent and when all baseline assessments have been completed. Participants will be allocated in a 1:1 ratio to either the intervention or usual care. Randomization will be performed using a validated, secure web-based randomization program (Sortition [Primary Care Trials Unity, University of Oxford]) provided by the University of Oxford Primary Care Clinical Trials Unit.

Allocation will be carried out with a nondeterministic minimization algorithm to ensure groups are balanced for important baseline prognostic and other factors: study site and age (<65 or ≥65 years); gender (male or female); duration of diabetes (<5 years or ≥5 years); and number of medications (<5 or ≥5). The allocated intervention will be implemented directly by the platform on which the digital health system is run. Apart from the qualitative research team and the engineering team, all other trials and health care staff were blinded to the treatment group. We determined that unblinding would not be required during the trial.

### Discontinuation of Intervention or Withdrawal From Trial

Participants can withdraw from the trial at any time. Participants can also choose to pause or stop the receipt of SMS text messages by sending an SMS text message or contacting the trial office by telephone or post. Serious unexpected adverse events related to the intervention are determined by the chief investigator (AF) and reported in line with the local procedures.

### Statistical Analysis

#### Power

A total sample size of 958 participants (479 per group) provides 92.5% power to detect a 4-percentage point change in cardiovascular risk of 4-percentage point change in risk (number needed to treat=25) based on an SD of 15% for cardiovascular risk derived from a primary care diabetes trial in patients with type 2 diabetes, in which reductions between 4% and 7% in estimated 10-year CVD risk were observed with statin treatment [33]. This estimate includes 15% inflation owing to clustering and 20% loss to follow-up at 92.5% power and 5% two-sided level of significance. The sample size also provides the power to detect changes in HbA_1c_ between groups of 4 mmol/mol based on an SD of 15 mmol/mol for patients newly starting glucose-lowering therapy [29]. This number of participants will also provide 80% power to detect an increase in the proportion of medication available from a baseline of 50% to 60.9%.

#### Analysis

The primary analysis population will include all randomized participants in the treatment arm to which they were assigned, regardless of the intervention received. Those found to be ineligible after randomization will be excluded from the analysis. For the primary and secondary outcomes, HbA_1c_ values will be included if they are between 3 and 12 months after randomization. The other data collected via notes review (cholesterol and blood pressure) will be included if between 6 weeks and 12 months post randomization.

Baseline variables will be presented by a randomized group using frequencies (with percentages) for binary and categorical variables and means (and SD) or medians (with lower and upper quartiles) for continuous variables.

The primary outcome will be analyzed using a multiple linear regression model. The model will adjust for baseline score, experience of diabetes education (yes or no), and minimization factors as fixed effects. Depending on the results of a preliminary exploration of the site, a mixed effect model will be used instead of a site fitted as a random effect. The adjusted difference in means between the 2 groups will be presented along with its associated 95% CI and *P* value.

Secondary continuous outcomes that are collected at 13, 26, and 52 weeks will be analyzed using a mixed effect model that includes a time × treatment interaction so that the treatment effect can be estimated at each time point; otherwise, the outcomes will be analyzed in a similar way to the primary outcome.

Similarly, binary outcomes measured at multiple time points will be analyzed using a generalized linear model (adjusting for the same factors listed earlier).

Missing data will be reported with reasons given where available, and the missing data pattern will be explored.

### Economic Analysis

The health economic analysis will be embedded in the clinical trial. The principal aim will be to assess the cost-effectiveness of the intervention as compared with usual care and will be accomplished by adopting an England NHS and Personal Social Services perspective, estimating total costs, and benefits expressed in QALYs. The intervention will be microcosted. The use of health care resources by participants of the trial will be estimated from self-reported questionnaires, hospital episode statistics, and Egton Medical Information Systems data and will be costed using current prices. Health utilities will be estimated using methods specified by the National Institute for Clinical Excellence at the time of analysis.

A health economic analysis plan will be agreed upon before the analysis, which will primarily be over the time horizon of the trial, and secondarily over a lifetime. QALYs over 1 year will be estimated directly from the clinical trial, and a trial-based incremental cost-effectiveness ratio will be calculated as the ratio of the difference in mean costs to the difference in QALYs. The joint uncertainty in costs and benefits will be considered through the application of bootstrapping and estimation of the cost-effectiveness acceptability curve.

If the intervention is determined to be clinically effective, costs and outcomes will be extrapolated using the UKPDS model [34]. Costs and outcomes accruing after the first year will be discounted according to the rate specified by the National Institute for Clinical Excellence at the time of analysis. The modeled extrapolation will be subject to probabilistic sensitivity analysis to characterize parameter uncertainty and present the probability of the adherence intervention being cost-effective. The health economic analysis will be reported according to the Consolidated Health Economic Evaluation Reporting Standards checklist [35].

### Process Evaluation

A process evaluation will be carried out in line with the MRC guidance on process evaluations [25] and an updated MRC framework [36]. The process evaluation will have quantitative and qualitative elements and will focus on (1) mechanisms (or theory) of change of the intervention, that is, how the intervention produces change in participants and (2) the impact of context on how the intervention works.

#### Participants

Participants who provide consent to take part in the embedded qualitative study will be purposefully sampled by characteristics including, but not limited to, age, gender, duration of diabetes, medication use (duration and number), current adherence, and familiarity with digital devices, with the aim of a maximum variation sample within the sampling framework of the trial.

Up to 60 participants will be recruited from the intervention group in 2 waves. The initial wave of up to 30 participants will be recruited for 2 interviews, at 1-month and 12-month follow-ups to explore any change after initial exposure to the intervention, and potential long-term change after 12 months. Up to an additional 30 participants will be recruited for interviews at 12 months following the analysis of the interim measures. This will allow purposive sampling based on any changes in psychological constructs.

Prompts in the interview guide will be informed by anticipated themes derived from the existing literature and our previous qualitative studies, but consistent with inductive qualitative research methodology, we will also invite stories of the experience of participants in the trial without imposing a strictly limited preconceived topic guide. Further areas for questioning will be added as necessary as new themes emerge from early interviews. Participants will be invited to share their views on how they engaged with the system, the messaging system content, and will be invited to describe how the system was implemented in daily life. This will support the identification of issues surrounding potential attrition.

All interviews will be audio-recorded (with consent), transcribed verbatim, and analyzed thematically [37]. We will involve people with diabetes closely in both the development of the interview guide and in testing and refining our interpretation of the data to ensure that the analysis is as relevant and credible to eventual users as possible.

#### Quantitative Data in the Process Analysis

**Figure 1:**
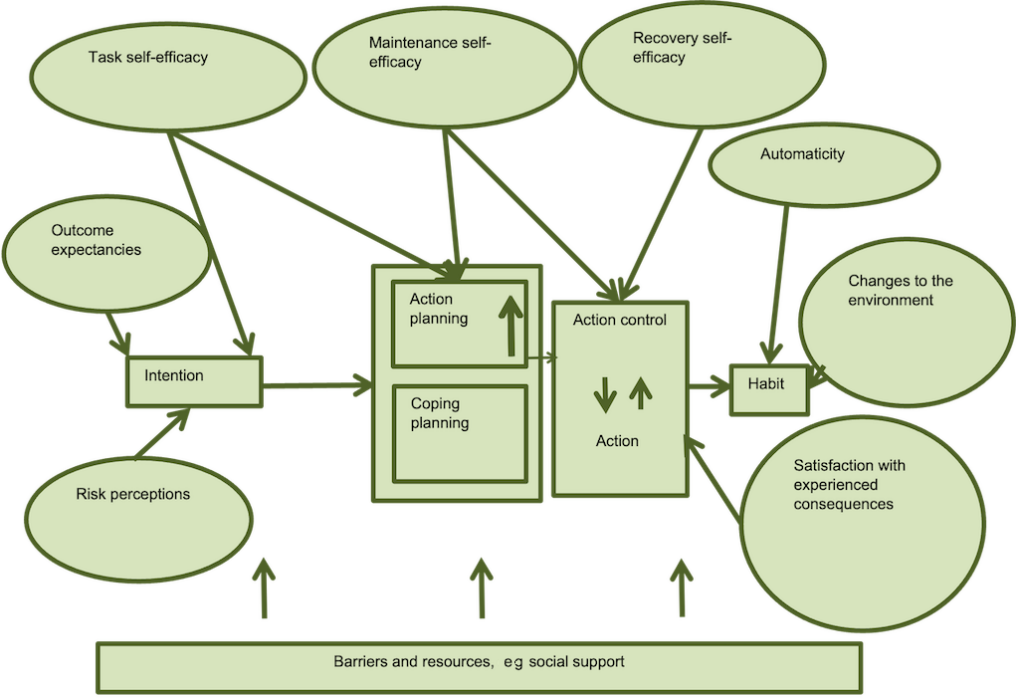
Process evaluation: logic model.

The trial will include brief questionnaires to assess the key behavior change constructs in which the messages are targeted at changing (Multimedia Appendix 2). An assessment will be made of the use of the system (messages received and responses) from routinely collected electronic data. In line with MRC guidance [36], we have developed a logic model (Figure 1) that indicates how the intervention will produce changes in behavior and thereby cardiovascular risk based on the Health Action Process Approach [38] and developmental work.

Three mediation analyses based on instrumental variable techniques [39-41] will be used to explore the extent to which changes in the behavior change constructs can explain any impact of the intervention on medication adherence measured by (1) MPR and (2) self-reported MARS and health measured by (3) the composite cardiovascular outcome. The constructs to be included are intention, action planning, coping planning, action control, habit, task self-efficacy, maintenance self-efficacy, recovery self-efficacy, automaticity, changes to the environment, satisfaction with experienced consequences, risk perceptions, outcome expectancies, social support, and patient activation. Relevant covariates will be included in the models, including age, gender, and index of multiple deprivation.

#### Qualitative Data in the Process Analysis

Interview data will be used to further explore the psychological mechanisms by probing what actions and feelings the intervention may result in, and the contextual factors that may influence how the intervention works for an individual. A content analysis of previous qualitative work has identified potential contextual factors and mechanisms that may influence how the SuMMiT-D intervention is working or not working. The connection between these and the effects of the intervention will be probed in these interviews. In accordance with recent guidelines related to potential measurement reactivity within trials, we have considered the effects these interviews may have on the trial outcomes and have taken steps to mitigate these [42]. All 52-week interviews will be conducted following quantitative data collection. The timing of the 4-week interviews has been chosen to allow exploration of initial changes in response to the messages while still leaving an appropriate gap between the interview and follow-up measurement (11 months). Sensitivity analyses will be considered as an option to examine the potential effects of being interviewed at the interim and final outcome points.

### Data Management

All trial data will be entered into electronic case report forms. The clinical database is built on the Research Electronic Data Capture, a secure, web-based application designed to support data capture for research studies [43].

### Ethics and Dissemination

The trial will be conducted according to the principles of the Declaration of Helsinki and in accordance with other relevant national guidelines, regulations, acts, and good clinical practice guidelines. The University of Oxford sponsors the trial.

The role of the Trial Steering Committee is taken on by the National Institute for Health Research Programme Steering Committee. The composition of the Trial Steering Committee is presented in Multimedia Appendix 3. The sponsor and funder determined this as a trial at low risk, and a Data Monitoring Committee has not been set up, with the Trial Steering Committee monitoring any problems arising. Ethical approval was obtained from the West of Scotland Research Ethics Committee 05.

The trial is sponsored by the University of Oxford, Clinical Trial and Research Governance Unit, Boundary Brook House, Headington, Oxford, United Kingdom. The sponsor and funder have no role in the study design, collection, management, analysis, and interpretation of data, writing of the report, and the decision to submit the report for publication.

### Data Sharing and Dissemination

Access to quantitative trial data will be made available following the publication of primary results through a publicly accessible repository. Qualitative data cannot be made openly available because of ethical concerns. Access conditions will be available from the repository. Trial findings will be made available on the International Standard Randomised Controlled Trial Number website as soon as possible and before publication. The participants will be informed of the findings. The findings will be communicated by conferences and social media.

### Dissemination Plan

The results of this trial will be submitted to a peer-reviewed journal for publication, through conference presentations, publications of process evaluation, and qualitative work, and on the SuMMiT-D trial website.

## Results

Recruitment to the SuMMiT-D trial began with the first participant randomized on March 23, 2021. General practices across England have agreed to identify and invite participants to participate in the study, and participants have been recruited from 42 general practices. The reporting of the trial is anticipated in 2024.

## Discussion

### Principal Findings

The SuMMiT-D trial is a large-scale randomized controlled trial that aims to estimate the clinical and cost-effectiveness of the SMS text messaging intervention compared with usual care. It addresses the need to develop and better understand scalable interventions that can address the continuing challenge of suboptimal medication adherence through the increasing capability of mobile phones and digital platforms.The trial is pragmatic in design and can provide information about the impact of brief messaging on people with diabetes with SMS text messages that have been systematically developed to use established BCTs. Although the trial focuses on selecting a population having type 2 diabetes, many, if not most, of these people will have other medical conditions; thus, it has broader applicability to a wide population of people who have type 2 diabetes in addition to other conditions rather than excluding those individuals.

### Conclusions

If effective, this intervention could help reduce the burden of complications and increase the costs associated with nonadherence. Alongside this trial, we are also looking at how this intervention, and those like it, could best be embedded in routine clinical care. This research could also offer a model for technology-based self-management support that could be extended to other aspects of diabetes care and other long-term conditions.

## Appendix

Multimedia Appendix 1 Standard Protocol Items: Template for Intervention, Description, and Replication checklist and Recommendations for Interventional Trials checklist.

Multimedia Appendix 2 Study-specific questionnaires and example text messages.

Multimedia Appendix 3 Study governance.

Multimedia Appendix 4 Peer review report.

## Abbreviations

BCT: behavior change technique
CVD: cardiovascular disease
EQ-5D-5L: EuroQol 5-dimension, 5-level
HbA_1c_: glycated hemoglobin
MARS: Medication Adherence Report Scale
MPR: medication possession ratio
MRC: Medical Research Council
NHS: National Health Service
NIHR: National Institute for Health Research
PPI: patient and public involvement
QALY: quality-adjusted life year
SuMMiT-D: Support Through Mobile Messaging and Digital Health Technology for Diabetes
UKPDS: United Kingdom Prospective Diabetes Study
