# Supplementary material for: Supporting People With Type 2 Diabetes in the Effective Use of Their Medicine Through Mobile Health Technology Integrated With Clinical Care to Reduce Cardiovascular Risk: Protocol for an Effectiveness and Cost-effectiveness Randomized Controlled Trial
Source: JMIR Res Protoc. 2022 Feb 21;11(2):e32918. doi: 10.2196/32918 (PMC8902673; doi:10.2196/32918)
Supplement: Multimedia Appendix 1 [file resprot_v11i2e32918_app1.docx]

Research Protocol

Title: Supporting people with type 2 diabetes in effective use of their medicine through mobile health technology integrated with clinical care to reduce cardiovascular risk (SuMMiT-D): an effectiveness and cost-effectiveness randomised control trial protocol

**Appendix 1 of supplementary material:**

**Contents**

TiDIER Statement

SPIRIT Checklist

**Appendix 1: Standard protocol items**

**Template for intervention, description, and replication (TiDIER) checklist**

| **TIDieR checklist for the Supporting people with type 2 diabetes in effective use of their medicine through mobile health technology integrated with clinical care (SuMMiT-D) trial** | | | | |
| --- | --- | --- | --- | --- |
| **1.** | **Brief name** | Supporting people with type 2 diabetes in effective use of their medicine through mobile health technology integrated with clinical care (SuMMiT-D) | | |
| **2.** | **Rationale or theory** | Systematic reviews of text messages used to support patients to adhere to treatment, and of mobile health interventions in diabetes, identify some effective interventions. There are a few trials testing the impact of brief messaging in type 2 diabetes, but they do not have systematically developed interventions based on theory and evidence and are at risk of bias.(1, 2) Recent trials of text-messaging for cardiovascular risk prevention and blood pressure lowering have shown clinically relevant changes in outcomes compared with usual care.(3, 4)  In addition, there is substantial evidence(5) that tailored interventions are more effective than generic interventions. Tailored interventions may be seen by recipients as more personally relevant, so they will be more likely to attend to, read, understand, and act on them. In addition, tailored interventions are designed to change determinants of the target behaviour that are relevant to particular individuals or to small subgroups of individuals; they therefore more precisely target the determinants of the individual’s behaviour.  SuMMiT-D (SUpport through Mobile Messaging and digital health Technology for Diabetes) is a programme of work comprised of three phases: formative work, a feasibility trial and a large scale, effectiveness randomised controlled trial of a mobile phone-based system intended to deliver brief, tailored, behaviour-change messages to people with type 2 diabetes focusing on use of medication, lifestyle and other aspects of diabetes management. In the formative work for this trial, we identified theoretical constructs and features of intervention content found to be associated with medication adherence in patients with type 2 diabetes and mapped these onto a standard taxonomy for behaviour change techniques (BCT), that is, active ingredients of interventions used to promote behavioural change.(6, 7) Based this work on the views of people with type 2 diabetes about the acceptability of this approach,(8) we then developed a large set of messages to target each BCT,(9) and examined which types of messages are most useful and easy to understand for people starting and taking an oral diabetes medicine and the extent to which it might be helpful for patients to decide on the types of messages they want to receive. We carried out a feasibility study,(10) and confirmed that the trial processes were acceptable and feasible,(11) and that the response to messages corresponded to that observed in the formative work.(12) | | |
|  |  | Usual care | Condition-specific tailored text messaging system plus usual care | |
|  |  |  | 1. Participants will be sent up to four automated text-messages per week with an average frequency of three per week relating to diabetes management and use of medicine. 2. The library of text-messages uses different behaviour change techniques to target health-related behaviour change relating to use of medicines, as well as messages targeting other aspects of diabetes care (including diet and exercise).. 3. Frequency of messages received using a particular type of behaviour change technique can be modified based on a participant's response to individual messages received.   The style of messages will be patient-centred and will encourage patients to seek further relevant information (including the use of links where possible to selected external websites e.g. Diabetes UK). | |
| **3.** | **Materials** | Available health materials on type 2 diabetes routinely provided by the health care service. | | |
| **4.** | **Procedures** |  | | 1. All participants were sent a system user guide by email. 2. Timing of messages selected by participant 3. Types of brief messages 4. Welcome messages (confirming sign-up) 5. Message with instructions about use of system 6. Brief treatment and lifestyle adherence support messages randomly selected from library (with rules that ensured individual messages were not repeated) and sent several times per week, each week for 6-months 7. Reminders of how to use the system to “Like” and “Dislike” messages, STOP and PAUSE sending of messages 8. Thank you message at end of study |
| **5.** | **Intervention provider** | Automated SMS text-message delivery platform using open-source software and third-party bulk SMS-delivery provider | | |
| **6.** | **Modes of delivery** | Intervention delivered via 160 characters SMS-text sent to individual participant’s own handset. Initial message sent to all enrolled participants is a “Welcome “message confirming sign-up, thereafter after automated messages sent to individual participants as per randomised allocation for 12 months. All trial participants receive infrequent (maximum every four weeks) non-health related messages sent to all participants for trial related purposes including to maintain participant interest in the trial. | | |
| **7.** | **Location where intervention occurred** | Community | | |
| **8.** | **Number of times intervention was delivered over what time period** |  | | Brief messages sent three (minimum) to four (maximum) times per week for 6-months |
| **9.** | **What, why, when, how intervention was personalised or adapted** |  | | 1. The times of day and days of the week for messages is selected by participant 2. The group (behavioural change technique) from which messages are sent is modified on the basis of the participant sending “likes” and “dislikes” as text-messages |
| **10.** | **Modifications during the trial** | Nil | | None |
| **11.** | **Planned intervention delivery** | Brief messages will be sent using an automated system independent of trial and clinical staff. Participants will be told that not everyone will be receiving the same messages. Participants will also be asked not to share their health messages with others. Intervention fidelity (receipt of messages and linkage to an individual) will be checked by response to the REGISTER request. Message delivery reports will be monitored throughout the trial to check the intervention is being delivered as planned. Messages not delivered (network unavailable etc.) will be resent up to three times. Where message delivery fails after three sequential attempts a failed message protocol will be initiated to track the participant and up-date their phone number if required. | | |
| **12.** | **Actual intervention delivery** | Will be monitored during trial, blind to allocated group | | |

1. Farmer AJ, McSharry J, Rowbotham S, McGowan L, Ricci-Cabello I, French DP. Effects of interventions promoting monitoring of medication use and brief messaging on medication adherence for people with Type 2 diabetes: a systematic review of randomized trials. Diabet Med. 2016;33(5):565-79.

2. Ricci-Cabello I, Bobrow K, Islam SMS, Chow CK, Maddison R, Whittaker R, et al. Examining Development Processes for Text Messaging Interventions to Prevent Cardiovascular Disease: Systematic Literature Review. JMIR Mhealth Uhealth. 2019;7(3):e12191.

3. Chow CK, Redfern J, Hillis GS, Thakkar J, Santo K, Hackett ML, et al. Effect of Lifestyle-Focused Text Messaging on Risk Factor Modification in Patients With Coronary Heart Disease. JAMA. 2015;314(12):1255.

4. Bobrow K, Farmer AJ, Springer D, Shanyinde M, Yu LM, Brennan T, et al. Mobile Phone Text Messages to Support Treatment Adherence in Adults With High Blood Pressure (SMS-Text Adherence Support [StAR]): A Single-Blind, Randomized Trial. Circulation. 2016;133(6):592-600.

5. Kassavou A, Sutton S. Automated telecommunication interventions to promote adherence to cardio-metabolic medications: meta-analysis of effectiveness and meta-regression of behaviour change techniques. Health Psychol Rev. 2018;12(1):25-42.

6. Michie S, Richardson M, Johnston M, Abraham C, Francis J, Hardeman W, et al. The behavior change technique taxonomy (v1) of 93 hierarchically clustered techniques: building an international consensus for the reporting of behavior change interventions. Ann Behav Med. 2013;46(1):81-95.

7. Long H, Bartlett YK, Farmer AJ, French DP. Identifying Brief Message Content for Interventions Delivered via Mobile Devices to Improve Medication Adherence in People With Type 2 Diabetes Mellitus: A Rapid Systematic Review. J Med Internet Res. 2019;21(1):e10421.

8. Bartlett YK, Newhouse N, Long HA, Farmer AJ, French DP. What do people with type 2 diabetes want from a brief messaging system to support medication adherence? Patient Prefer Adherence. 2019;13:1629-40.

9. Bartlett YK, Farmer A, Rea R, French DP. Use of Brief Messages Based on Behavior Change Techniques to Encourage Medication Adherence in People With Type 2 Diabetes: Developmental Studies. J Med Internet Res. 2020;22(5):e15989.

10. Farmer A, Allen J, Bartlett K, Bower P, Chi Y, French D, et al. Supporting people with type 2 diabetes in effective use of their medicine through mobile health technology integrated with clinical care (SuMMiT-D Feasibility): a randomised feasibility trial protocol. BMJ Open. 2019;9(12):e033504.

11. Chi Y, Velardo C, Allen J, Robinson S, Riga E, Judge D, et al. System Architecture for "Support Through Mobile Messaging and Digital Health Technology for Diabetes" (SuMMiT-D): Design and Performance in Pilot and Randomized Controlled Feasibility Studies. JMIR Formative Research. 2021;5(3):e18460.

12. Bartlett YK, Kenning C, Crosland J, Newhouse N, Miles LM, Williams V, et al. Understanding acceptability in the context of text messages to encourage medication adherence in people with type 2 diabetes. BMC Health Services Research. 2021;21(1).

# Reporting checklist for protocol of a clinical trial.

Based on the SPIRIT guidelines.

Supporting people with type 2 diabetes in effective use of their medicine through mobile health technology integrated with clinical care to reduce cardiovascular risk (SuMMiT-D): an effectiveness and cost-effectiveness randomised control trial protocol

|  |  | Reporting Item | Page Number |
| --- | --- | --- | --- |
| **Administrative information** |  |  |  |
| Title | [#1](https://www.goodreports.org/spirit/info/#1) | Descriptive title identifying the study design, population, interventions, and, if applicable, trial acronym | 1 |
| Trial registration | [#2a](https://www.goodreports.org/spirit/info/#2a) | Trial identifier and registry name. If not yet registered, name of intended registry | 3 |
| Trial registration: data set | [#2b](https://www.goodreports.org/spirit/info/#2b) | All items from the World Health Organization Trial Registration Data Set | 3 |
| Protocol version | [#3](https://www.goodreports.org/spirit/info/#3) | Date and version identifier | 1 |
| Funding | [#4](https://www.goodreports.org/spirit/info/#4) | Sources and types of financial, material, and other support | After 17 |
| Roles and responsibilities: contributorship | [#5a](https://www.goodreports.org/spirit/info/#5a) | Names, affiliations, and roles of protocol contributors | 17 and following |
| Roles and responsibilities: sponsor contact information | [#5b](https://www.goodreports.org/spirit/info/#5b) | Name and contact information for the trial sponsor | 17 |
| Roles and responsibilities: sponsor and funder | [#5c](https://www.goodreports.org/spirit/info/#5c) | Role of study sponsor and funders, if any, in study design; collection, management, analysis, and interpretation of data; writing of the report; and the decision to submit the report for publication, including whether they will have ultimate authority over any of these activities | 17 |
| Roles and responsibilities: committees | [#5d](https://www.goodreports.org/spirit/info/#5d) | Composition, roles, and responsibilities of the coordinating centre, steering committee, endpoint adjudication committee, data management team, and other individuals or groups overseeing the trial, if applicable (see Item 21a for data monitoring committee) | 17 and appendix |
| **Introduction** |  |  |  |
| Background and rationale | [#6a](https://www.goodreports.org/spirit/info/#6a) | Description of research question and justification for undertaking the trial, including summary of relevant studies (published and unpublished) examining benefits and harms for each intervention | 4 |
| Background and rationale: choice of comparators | [#6b](https://www.goodreports.org/spirit/info/#6b) | Explanation for choice of comparators | 5 |
| Objectives | [#7](https://www.goodreports.org/spirit/info/#7) | Specific objectives or hypotheses | 5 |
| Trial design | [#8](https://www.goodreports.org/spirit/info/#8) | Description of trial design including type of trial (eg, parallel group, crossover, factorial, single group), allocation ratio, and framework (eg, superiority, equivalence, non-inferiority, exploratory) | 6 |
| **Methods: Participants, interventions, and outcomes** |  |  |  |
| Study setting | [#9](https://www.goodreports.org/spirit/info/#9) | Description of study settings (eg, community clinic, academic hospital) and list of countries where data will be collected. Reference to where list of study sites can be obtained | 6 |
| Eligibility criteria | [#10](https://www.goodreports.org/spirit/info/#10) | Inclusion and exclusion criteria for participants. If applicable, eligibility criteria for study centres and individuals who will perform the interventions (eg, surgeons, psychotherapists) | 9 |
| Interventions: description | [#11a](https://www.goodreports.org/spirit/info/#11a) | Interventions for each group with sufficient detail to allow replication, including how and when they will be administered | 7 plus TiDierR checklist |
| Interventions: modifications | [#11b](https://www.goodreports.org/spirit/info/#11b) | Criteria for discontinuing or modifying allocated interventions for a given trial participant (eg, drug dose change in response to harms, participant request, or improving / worsening disease) | 11 |
| Interventions: adherance | [#11c](https://www.goodreports.org/spirit/info/#11c) | Strategies to improve adherence to intervention protocols, and any procedures for monitoring adherence (eg, drug tablet return; laboratory tests) | 11 |
| Interventions: concomitant care | [#11d](https://www.goodreports.org/spirit/info/#11d) | Relevant concomitant care and interventions that are permitted or prohibited during the trial | N/A |
| Outcomes | [#12](https://www.goodreports.org/spirit/info/#12) | Primary, secondary, and other outcomes, including the specific measurement variable (eg, systolic blood pressure), analysis metric (eg, change from baseline, final value, time to event), method of aggregation (eg, median, proportion), and time point for each outcome. Explanation of the clinical relevance of chosen efficacy and harm outcomes is strongly recommended | 7 |
| Participant timeline | [#13](https://www.goodreports.org/spirit/info/#13) | Time schedule of enrolment, interventions (including any run-ins and washouts), assessments, and visits for participants. A schematic diagram is highly recommended (see Figure) | 9/10 |
| Sample size | [#14](https://www.goodreports.org/spirit/info/#14) | Estimated number of participants needed to achieve study objectives and how it was determined, including clinical and statistical assumptions supporting any sample size calculations | 12 |
| Recruitment | [#15](https://www.goodreports.org/spirit/info/#15) | Strategies for achieving adequate participant enrolment to reach target sample size | 8 |
| **Methods: Assignment of interventions (for controlled trials)** |  |  |  |
| Allocation: sequence generation | [#16a](https://www.goodreports.org/spirit/info/#16a) | Method of generating the allocation sequence (eg, computer-generated random numbers), and list of any factors for stratification. To reduce predictability of a random sequence, details of any planned restriction (eg, blocking) should be provided in a separate document that is unavailable to those who enrol participants or assign interventions | 11 |
| Allocation concealment mechanism | [#16b](https://www.goodreports.org/spirit/info/#16b) | Mechanism of implementing the allocation sequence (eg, central telephone; sequentially numbered, opaque, sealed envelopes), describing any steps to conceal the sequence until interventions are assigned | 11 |
| Allocation: implementation | [#16c](https://www.goodreports.org/spirit/info/#16c) | Who will generate the allocation sequence, who will enrol participants, and who will assign participants to interventions | 11 |
| Blinding (masking) | [#17a](https://www.goodreports.org/spirit/info/#17a) | Who will be blinded after assignment to interventions (eg, trial participants, care providers, outcome assessors, data analysts), and how | 11 |
| Blinding (masking): emergency unblinding | [#17b](https://www.goodreports.org/spirit/info/#17b) | If blinded, circumstances under which unblinding is permissible, and procedure for revealing a participant’s allocated intervention during the trial | 11 |
| **Methods: Data collection, management, and analysis** |  |  |  |
| Data collection plan | [#18a](https://www.goodreports.org/spirit/info/#18a) | Plans for assessment and collection of outcome, baseline, and other trial data, including any related processes to promote data quality (eg, duplicate measurements, training of assessors) and a description of study instruments (eg, questionnaires, laboratory tests) along with their reliability and validity, if known. Reference to where data collection forms can be found, if not in the protocol | 9/10 |
| Data collection plan: retention | [#18b](https://www.goodreports.org/spirit/info/#18b) | Plans to promote participant retention and complete follow-up, including list of any outcome data to be collected for participants who discontinue or deviate from intervention protocols | 9/10/11 |
| Data management | [#19](https://www.goodreports.org/spirit/info/#19) | Plans for data entry, coding, security, and storage, including any related processes to promote data quality (eg, double data entry; range checks for data values). Reference to where details of data management procedures can be found, if not in the protocol | 16 |
| Statistics: outcomes | [#20a](https://www.goodreports.org/spirit/info/#20a) | Statistical methods for analysing primary and secondary outcomes. Reference to where other details of the statistical analysis plan can be found, if not in the protocol | 12 |
| Statistics: additional analyses | [#20b](https://www.goodreports.org/spirit/info/#20b) | Methods for any additional analyses (eg, subgroup and adjusted analyses) | 12/13 |
| Statistics: analysis population and missing data | [#20c](https://www.goodreports.org/spirit/info/#20c) | Definition of analysis population relating to protocol non-adherence (eg, as randomised analysis), and any statistical methods to handle missing data (eg, multiple imputation) | 12/13 |
| **Methods: Monitoring** |  |  |  |
| Data monitoring: formal committee | [#21a](https://www.goodreports.org/spirit/info/#21a) | Composition of data monitoring committee (DMC); summary of its role and reporting structure; statement of whether it is independent from the sponsor and competing interests; and reference to where further details about its charter can be found, if not in the protocol. Alternatively, an explanation of why a DMC is not needed | 17 |
| Data monitoring: interim analysis | [#21b](https://www.goodreports.org/spirit/info/#21b) | Description of any interim analyses and stopping guidelines, including who will have access to these interim results and make the final decision to terminate the trial | N/A |
| Harms | [#22](https://www.goodreports.org/spirit/info/#22) | Plans for collecting, assessing, reporting, and managing solicited and spontaneously reported adverse events and other unintended effects of trial interventions or trial conduct | 12 |
| Auditing | [#23](https://www.goodreports.org/spirit/info/#23) | Frequency and procedures for auditing trial conduct, if any, and whether the process will be independent from investigators and the sponsor | N/A |
| **Ethics and dissemination** |  |  |  |
| Research ethics approval | [#24](https://www.goodreports.org/spirit/info/#24) | Plans for seeking research ethics committee / institutional review board (REC / IRB) approval | 2 |
| Protocol amendments | [#25](https://www.goodreports.org/spirit/info/#25) | Plans for communicating important protocol modifications (eg, changes to eligibility criteria, outcomes, analyses) to relevant parties (eg, investigators, REC / IRBs, trial participants, trial registries, journals, regulators) | N/A |
| Consent or assent | [#26a](https://www.goodreports.org/spirit/info/#26a) | Who will obtain informed consent or assent from potential trial participants or authorised surrogates, and how (see Item 32) | 9 |
| Consent or assent: ancillary studies | [#26b](https://www.goodreports.org/spirit/info/#26b) | Additional consent provisions for collection and use of participant data and biological specimens in ancillary studies, if applicable | N/A |
| Confidentiality | [#27](https://www.goodreports.org/spirit/info/#27) | How personal information about potential and enrolled participants will be collected, shared, and maintained in order to protect confidentiality before, during, and after the trial | 16 |
| Declaration of interests | [#28](https://www.goodreports.org/spirit/info/#28) | Financial and other competing interests for principal investigators for the overall trial and each study site | After 18 |
| Data access | [#29](https://www.goodreports.org/spirit/info/#29) | Statement of who will have access to the final trial dataset, and disclosure of contractual agreements that limit such access for investigators | 14 |
| Ancillary and post trial care | [#30](https://www.goodreports.org/spirit/info/#30) | Provisions, if any, for ancillary and post-trial care, and for compensation to those who suffer harm from trial participation | N/A |
| Dissemination policy: trial results | [#31a](https://www.goodreports.org/spirit/info/#31a) | Plans for investigators and sponsor to communicate trial results to participants, healthcare professionals, the public, and other relevant groups (eg, via publication, reporting in results databases, or other data sharing arrangements), including any publication restrictions | 14 |
| Dissemination policy: authorship | [#31b](https://www.goodreports.org/spirit/info/#31b) | Authorship eligibility guidelines and any intended use of professional writers | N/A |
| Dissemination policy: reproducible research | [#31c](https://www.goodreports.org/spirit/info/#31c) | Plans, if any, for granting public access to the full protocol, participant-level dataset, and statistical code | 14 |
| **Appendices** |  |  |  |
| Informed consent materials | [#32](https://www.goodreports.org/spirit/info/#32) | Model consent form and other related documentation given to participants and authorised surrogates | Appendix |
| Biological specimens | [#33](https://www.goodreports.org/spirit/info/#33) | Plans for collection, laboratory evaluation, and storage of biological specimens for genetic or molecular analysis in the current trial and for future use in ancillary studies, if applicable | N/A |

None The SPIRIT checklist is distributed under the terms of the Creative Commons Attribution License CC-BY-ND 3.0. This checklist can be completed online using <https://www.goodreports.org/>, a tool made by the [EQUATOR Network](https://www.equator-network.org) in collaboration with [Penelope.ai](https://www.penelope.ai)

Appenxix 3: Study Specific Questionaires

Examples of text messages and linked behaviour change techniques

| Target and category of message | Behaviour change technique/ Belief or concern | Example messages |
| --- | --- | --- |
| Medication adherence, BCT | Action Planning | Plan when, where and how you are going to take your medication. |
| Medication adherence, BCT | Verbal persuasion about capability | If you are struggling with your diabetes tablets then don't worry, you will be able to master it in time.  You will get on top of it.c |
| Medication adherence, BCT | Prompts/ cues | It can be difficult to remember to take your tablets. Why not set an alarm to remind you to take them? |
| Medication adherence, BCT | Self-monitoring | Find a way to split your tablets into days so you notice when you have forgotten to take your tablets |
| Medication adherence, BCT | 3.2 Social support (practical) | How often do you forget to take your tablets? Take control. Ask your friends and family members to help. Their reminders could help you to improve your diabetes |
| Medication adherence, BCT | Mental rehearsal of successful performance | Visualise in detail how you will take your tablets tomorrow. This will make it easier when you actually take them |
| Medication adherence, BCT | Social support (emotional) | If you're not taking your tablets as often as you should, try discussing your feelings with someone. |
| Medication adherence, BCT | Mental rehearsal of successful performance | Think about situations where taking tablets was easy.  How could you make your everyday tablet taking like this? |
| Medication adherence, beliefs and concerns | Healthcare system related concerns | Lots of questions? Check who the best person to see might be |
| Diet management | Signposting | Stuck for new ideas? You can search recipes for mains, desserts and snacks online at [Diabetes.org.uk](http://diabetes.org.uk/) |
