# Supplementary material for: Supporting People With Type 2 Diabetes in the Effective Use of Their Medicine Through Mobile Health Technology Integrated With Clinical Care to Reduce Cardiovascular Risk: Protocol for an Effectiveness and Cost-effectiveness Randomized Controlled Trial
Source: JMIR Res Protoc. 2022 Feb 21;11(2):e32918. doi: 10.2196/32918 (PMC8902673; doi:10.2196/32918)
Supplement: Multimedia Appendix 2 [file resprot_v11i2e32918_app2.docx]

Research Protocol

Title: Supporting people with type 2 diabetes in effective use of their medicine through mobile health technology integrated with clinical care to reduce cardiovascular risk (SuMMiT-D): an effectiveness and cost-effectiveness randomised control trial protocol

**Appendix 2 of supplementary materials:**

**Contents**

Hypothesised Mediators Questionaire

Resource Use Questionaire

Attitudes to diabetes

Examples of text messages

**Appendix 2: Study Specific Questionaires and examples of SMS text messages**

Examples of text messages and linked behaviour change techniques

| Target and category of message | Behaviour change technique/ Belief or concern | Example messages |
| --- | --- | --- |
| Medication adherence, BCT | Action Planning | Plan when, where and how you are going to take your medication. |
| Medication adherence, BCT | Verbal persuasion about capability | If you are struggling with your diabetes tablets then don't worry, you will be able to master it in time.  You will get on top of it.c |
| Medication adherence, BCT | Prompts/ cues | It can be difficult to remember to take your tablets. Why not set an alarm to remind you to take them? |
| Medication adherence, BCT | Self-monitoring | Find a way to split your tablets into days so you notice when you have forgotten to take your tablets |
| Medication adherence, BCT | 3.2 Social support (practical) | How often do you forget to take your tablets? Take control. Ask your friends and family members to help. Their reminders could help you to improve your diabetes |
| Medication adherence, BCT | Mental rehearsal of successful performance | Visualise in detail how you will take your tablets tomorrow. This will make it easier when you actually take them |
| Medication adherence, BCT | Social support (emotional) | If you're not taking your tablets as often as you should, try discussing your feelings with someone. |
| Medication adherence, BCT | Mental rehearsal of successful performance | Think about situations where taking tablets was easy.  How could you make your everyday tablet taking like this? |
| Medication adherence, beliefs and concerns | Healthcare system related concerns | Lots of questions? Check who the best person to see might be |
| Diet management | Signposting | Stuck for new ideas? You can search recipes for mains, desserts and snacks online at [Diabetes.org.uk](http://diabetes.org.uk/) |
