# Supplementary material for: Supporting People With Type 2 Diabetes in the Effective Use of Their Medicine Through Mobile Health Technology Integrated With Clinical Care to Reduce Cardiovascular Risk: Protocol for an Effectiveness and Cost-effectiveness Randomized Controlled Trial
Source: JMIR Res Protoc. 2022 Feb 21;11(2):e32918. doi: 10.2196/32918 (PMC8902673; doi:10.2196/32918)
Supplement: Multimedia Appendix 3 [file resprot_v11i2e32918_app3.docx]

Research Protocol

Title: Supporting people with type 2 diabetes in effective use of their medicine through mobile health technology integrated with clinical care to reduce cardiovascular risk (SuMMiT-D): an effectiveness and cost-effectiveness randomised control trial protocol

**Appendix 3 of of supplementary material:**

**Study Governance**

List of practices involved

List of Trial Steeering Committee members

**Appendix 1 Study Governance**

**List of practices and lead primary care physicians**

Ashton Medical Group Dr Omair Razzaq

The Sides Medical Practice Dr Laurence Cribbin

Peterloo Medical Centre, Dr Imran Ghafoor

Marine Lake Medical Practice Dr James Perry

Vauxhall Primary Health Care Dr David Lewis

St Georges Medical Centre Dr Rebecca Sinfield

Station House Surgery Dr David Cowling

Pendle Medical Partnership Dr Tim Smith

Queen Square Medical Practice Dr Rachel Woolley

Heswall & Pensby Group Practice Dr Stephen Forster

Lancaster Medical Practice Dr Michael Wong

Pendle View Medical Centre Dr Umesh Chauhan

Devonshire Green Medical Centre Dr Kieran Brown

Cartmel Surgery Dr Julie Colclough

Ashfields Primary Care Centre Dr Neil Paul

Kiltearn Medical Centre Dr Carolyn Paul

Oakenhurst Medical Practice Dr Amar Ali

Bay Medical Group Dr Russell Kelton

Darwen Healthcare Dr Mammen Ninan

Eynsham Medical Group Ian Binnian

Church Street Practice Matthew Gaw

Iver Medical Centre Neetul Shah

Banbury Cross Health Centre James Kennard

Wing Surgery Chris Davies

Norden House Surgery Chris Davies

Whitchurch Surgery Chris Davies

Liphook and Liss Surgery Anna Lalonde

Park and St. Francis Surgery Sam Glanville

Westlands Medical Centre Helen Pandya

Andover Health Centre Lucy Allen

The Bosmere Medical Practice Dirk Konig

Mid Devon Medical Practice Will Edney

Chilcote Surgery E. Funnell

The Swan Practice Chris Davies

Crewkerne Health Centre Christopher Krasucki

Cheriton Bishop Surgery James Hayter

Lawrence Hill Health Centre Jack Ogden

Phoenix Health Group Naomi Vernon

West Walk Surgery Sam Davies

Rendcomb Surgery Clare Henderson

Cotswold Medical Practice Nicholas Hodgkins

Greenway Community Practice Liz Grimshaw

Mendip Vale Medical Practice Richard Reed

**List of Trial Steering Committee members**

*Internal team members*

Prof Andrew Farmer

Prof Peter Bower

Prof Ly-Mee Yu

*External members*

Prof Kamlesh Khunti (Chair)

Mr Steve England

Prof Falko Sniehotta

Prof Christopher Weir
