# Supplementary material for: Supporting People With Type 2 Diabetes in the Effective Use of Their Medicine Through Mobile Health Technology Integrated With Clinical Care to Reduce Cardiovascular Risk: Protocol for an Effectiveness and Cost-effectiveness Randomized Controlled Trial
Source: JMIR Res Protoc. 2022 Feb 21;11(2):e32918. doi: 10.2196/32918 (PMC8902673; doi:10.2196/32918)
Supplement: Multimedia Appendix 4 [file resprot_v11i2e32918_app4.pdf]

# NATIONAL INSTITUTE FOR HEALTH RESEARCH

# PROGRAMME GRANTS FOR APPLIED RESEARCH

## Assessment Template

| | |
|---|---|
| **Proposal Reference** | RP-PG-1214-20003 |
| **Lead Applicant** | Professor Andrew Farmer |
| **Lead NHS Trust** | Oxford Health NHS Foundation Trust |
| **Programme Title** | Supporting people with type 2 diabetes in effective use of their medicine through a system comprising mobile health technology integrated with clinical care |
| **Sub-Panel and Chair** | A; Professor [redacted] |

The Sub-Panel's assessment of the proposal against the selection criteria was as follows:

**1. The track-record of the applicants in conducting high-quality applied health research, as judged by publication output and previous research funding**

The strength and track record of the team was acknowledged.

**2. The relevance of the proposed research to the priorities and needs of the NHS**

The proposal's relevance and importance was recognised.

**3. The likelihood of significant benefit to the NHS and patients within three to five years of the end of the programme**

As presented, the proposal offered a moderate likelihood of delivering benefit to patients and the NHS within 3 – 5 years of the funding period, especially as the intervention was particularly under developed.

**4. The quality of the proposal**

The sub-panel raised the following points:

- The proposal had adapted well to previous feedback and was now stronger.
- The overall arrangements for patient and public involvement (PPI) were welcomed although there remained concerns from some sub-panel members that co-applicant [redacted] might be unrepresentative of the target population given his background ([redacted]) and longstanding association with the Principal Investigator. Augmentation of the patient advisory group to achieve more diversity was recommended.
- The sub-panel viewed 'personalisation' of text messages as crucial to the

success of the programme and they wished to know what other approaches to tailoring would be used in addition to the data linkage work, particularly if work package (WP) 2.3 could not be successfully delivered.

- Related to the above, it was agreed that the feasibility trial should also be used to examine the feasibility of the use of electronic records to link to messages in real time as well as to test outcomes data collection.
- The sub-panel emphasised the importance of exploring a person's concept of personalisation and what that meant for them in the early phase of the programme.
- The importance of fully drawing and building on the existing literature in this area (both type 2 diabetes (T2DM) and other conditions) was emphasised. This was particularly pertinent for WP 4 – further elaboration of how it would add to existing qualitative studies exploring mobile based support would be beneficial.
- WP 5 was still considered to be a little underspecified and should be described more fully, notably, how the data collected throughout the study would be brought together (in WP5) to more explicitly deliver outputs relating to further implementation and scalability.
- Proposals to make the intervention accessible to BME community members, especially those who do not readily read English (a particular concern since T2DM is more common amongst those of SE Asian origin) should be described - there appeared to be no budget or time allocated to whether and how messages could be translated into other languages, or whether they could be rendered into text messages for languages which do not use the Roman alphabet. Sub-panel members agreed that addressing issues of language and literacy would not only improve the proposal but also benefit the wider health services research community, where this is a problem that needs further investigation.
- The criteria for progression to the main trial were considered to be a little too stringent, more nuanced stop/go rules should be considered.
- The health economic component was welcomed although its role in the early parts of the programme should be clarified further as it still remained a little unclear.
- It should be clarified how QALYs would be calculated – from existing data sets?
- Some elucidation of the core constructs of NPT and how they might be worked into the design, data collection and collation of data was needed as the core constructs of the theory identified in the proposal did not seem to match NPT.
- Sub-panel members emphasised the importance of exploring the grey literature, especially when considering barriers to implementation within the NHS.
- Testing the draft messages for comprehensibility, and whether users understood the messages as intended (perhaps using paraphrasing techniques) as well as their 'credibility, acceptability and feasibility' would be beneficial.
- Given the importance of the theory to the proposed intervention, consideration should be given to analysing the data in ways that allow an understanding of whether different types of messages differentially affect the behaviours they could be expected to impact on (e.g. whether practical messages were more likely to lead to improved appointment attendance?).
- Although improved, the plain English summary would benefit from further simplification. The suggestions for improvement offered by reviewer 9 resonated with sub-panel members and hence should be considered.
- It was highlighted that the trial was not a CTIMP; hence Clinical Trial Authorisation from MHRA would not be required.
- Sub-panel members commented that the 'Flowhealth' (https://www.flowhealth.com/) website could usefully inform the proposal as it is concerned with personalised e-health.
- Some concerns around potential intellectual property (IP) were identified which

must be addressed.

## 5. The value for money provided by the proposal

It was agreed that overall, the proposal had the potential to offer value for money.

However, a number of specific finance concerns were identified which will be sent by the NIHR CCF contracts and finance team for attention.

## 6. Conclusions and Recommendations

The sub-panel recommended that initial funding be made available for 36 months to undertake the early parts of the programme, on the condition that the concerns outlined in sections 4 and 5 above are satisfactorily addressed.

A report would be expected by the NIHR (at 30 months) describing progress of the programme, with particular emphasis on the results of the feasibility work and the protocol for the definitive trial proposed; if satisfactory, the remaining funds would be released.

The applicants would need to provide a written response to these recommendations and concerns as soon as possible, and not later than [redacted], before funding could be awarded.

The response should be detailed in the template provided and should form no more than 6 pages. An annex of no more than 10 pages may be included.

Response to the finance and IP report should be detailed in the appropriate template provided and should form no more than 6 pages. Annexes are not permitted.
